# Personal

**Published:** 1894-04

**Authors:** 


### Personal.

DR. JOHN FARMER WINN, of Richmond, Va., has resigned the professorship of diseases of the nervous system, and has been appointed clinical professor and demonstrator of obstetrics in the University College of Medicine, Richmond. Dr. Winn is well fitted for his new chair, both by inclination and special training. His offices are at 109 West Grace street, Richmond, Va.

---

DR. MAUD J. FRYE, of Buffalo, has been appointed clinical lecturer on diseases of children in the Buffalo University Medical School. Dr. Frye is admirably equipped for the work in question, and, as the first woman to receive an appointment to the college staff, enjoys a most conspicuous honor.

---

DR. C. M. DANIELS, of Buffalo, has removed from 868 Main street to 315 Jersey street, corner Plymouth avenue. Hours: 2 to 4 o'clock P. M. Practice confined exclusively to surgery and gynecology.

---

DR. C. S. SIEGFRIED, of Buffalo, has removed from 149 Franklin street to 280 Franklin street. Hours: 8 to 10 A. M., 1 to 3 and 7 to 8 P. M.

---

DR. M. M. BROWN, of Elmira, has removed to Buffalo, and established his office and residence at 891 Niagara street.

